# C-reactive protein-to-albumin ratio as a novel prognostic biomarker for long-term mortality in pericarditis: a real-world study

**DOI:** 10.1186/s12872-025-05464-3

**Published:** 2025-12-30

**Authors:** Lingyu Mi, Ishan Lakhani, Sharen Lee, Wing Tak Wong, Gary Tse, Fang Fang

**Affiliations:** 1 Department of Structural Heart Disease, Fuwai Hospital & National Center for Cardiovascular Disease, Key Laboratory of Innovative Cardiovascular Devices, Chinese Academy of Medical Sciences & Peking Union Medical College, National Health Commission Key Laboratory of Cardiovascular Regeneration Medicine, National Clinical Research Center for Cardiovascular Diseases, Beilishi Road No. 167, Xicheng District, Beijing, 100037 China; 2 Department of Medicine, Queen Elizabeth Hospital, Hong Kong, China; 3 School of Life Sciences, The Chinese University of Hong Kong, Hong Kong, China; 4 CUHK Graduate School, The Chinese University of Hong Kong, Hong Kong, China; 5 Fuwai Yunnan Hospital, Chinese Academy of Medical Sciences/Affiliated Cardiovascular Hospital of Kunming Medical University, Kunming, China

**Keywords:** C-reactive protein–to–albumin ratio, Pericarditis, Inflammation, Nutritional status, All-cause mortality, Prognostic biomarker, Risk stratification

## Abstract

**Background:**

Pericarditis is a heterogeneous inflammatory condition with variable clinical outcomes. Although traditional inflammatory biomarkers such as C-reactive protein (CRP) and erythrocyte sedimentation rate (ESR) are routinely used for diagnosis and monitoring, they do not fully capture the interplay between inflammation, hepatic synthetic function, and nutritional status. The CRP–to–albumin ratio (CAR), a composite index integrating these components, has shown prognostic value in several cardiovascular disorders. However, its significance in pericarditis remains unknown.

**Methods:**

This was a real-world retrospective cohort study of adult patients hospitalized for pericarditis between January 1st, 2005 to December 31st, 2019 from a single tertiary centre. CAR was calculated as CRP (mg/L) divided by serum albumin (g/L) and categorized into quartiles. The primary outcome was all-cause mortality. Associations were examined using Cox proportional hazards models, restricted cubic splines (RCS), and segmented Cox regression, with prespecified sensitivity analyses using alternative adjustment strategies and modeling specifications.

**Results:**

A total of 546 patients (mean age, 59.2 ± 16.4 years; 56.8% men) were analyzed. During a median follow-up of 64 months, 239 deaths (43.8%) occurred. Higher CAR quartiles were associated with progressively increased mortality (log-rank *P* < 0.001). In multivariable Cox models adjusting for demographics and comorbidities, each unit increase in CAR conferred a 5% higher mortality risk (hazard ratio [HR], 1.05; 95% confidence interval [CI], 1.01–1.10; *P* = 0.031). Compared with the lowest quartile, adjusted HRs for mortality were 2.04 (95% CI, 1.34–3.09), 2.21 (95% CI, 1.47–3.32), and 1.93 (95% CI, 1.26–2.95) across quartiles 2–4 (P for trend = 0.004). RCS and segmented Cox analyses demonstrated a nonlinear relationship with a threshold near CAR = 0.33—below which mortality risk increased sharply and plateaued thereafter, which should be interpreted as exploratory given limited event density at very low CAR values. The overall association remained directionally consistent in sensitivity analyses including alternative knot specifications and approaches to address extreme values. Associations were consistent across age, sex, hypertension, and malignancy subgroups.

**Conclusions:**

CAR independently predicted long-term all-cause mortality in patients hospitalized for pericarditis, exhibiting a nonlinear dose–response pattern. CAR represents a simple, inexpensive, and readily available biomarker that integrates inflammatory and nutritional status, with robust findings across multiple sensitivity analyses, offering incremental prognostic value beyond traditional risk factors.

**C-reactive protein-to-albumin ratio predicts long-term all-cause mortality in pericarditis:**

**Figure:**
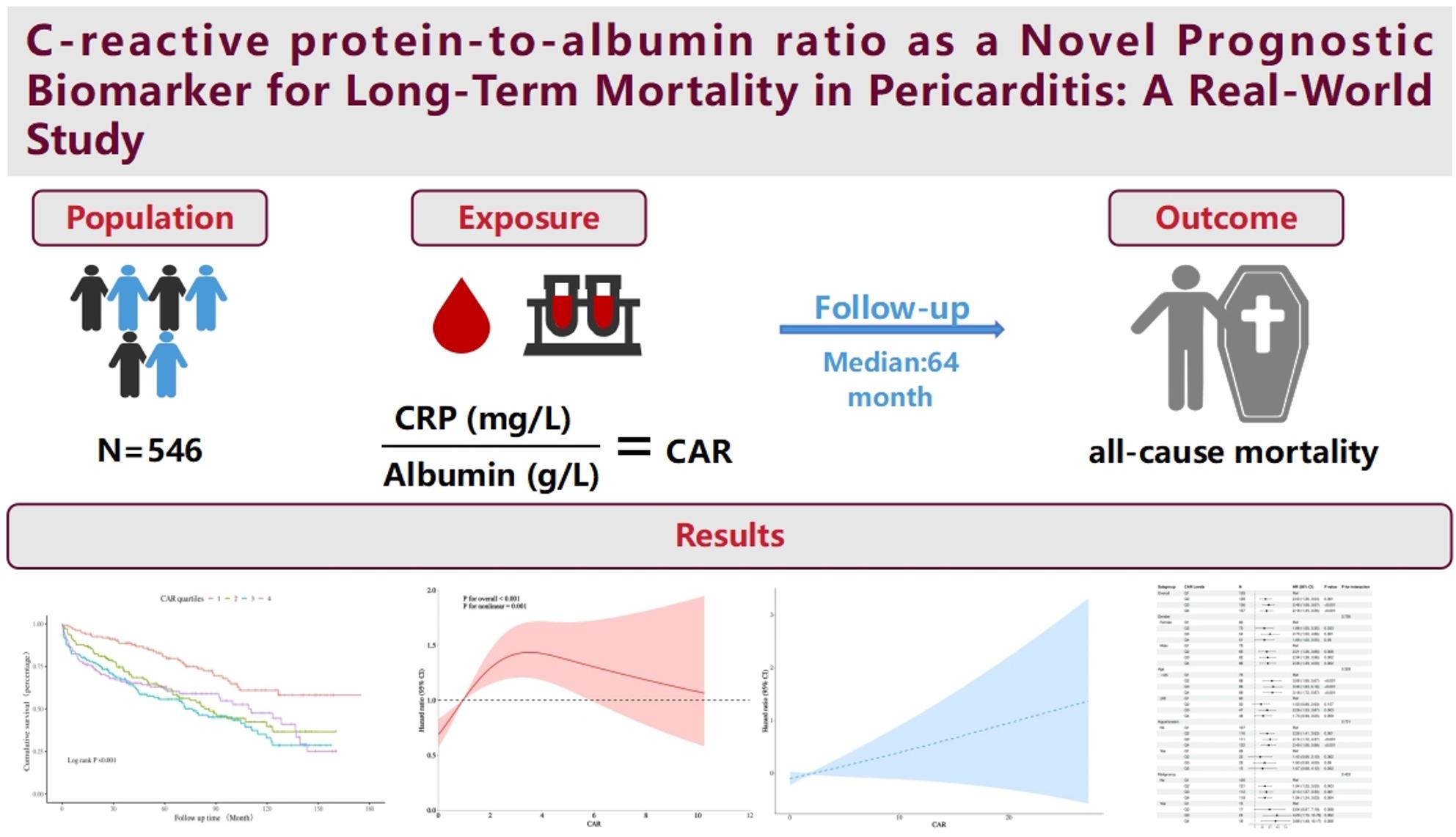
Study design and main findings: higher CRP-to-albumin ratio is associated with increased long-term all-cause mortality in pericarditis

**Supplementary Information:**

The online version contains supplementary material available at 10.1186/s12872-025-05464-3.

## Introduction

Pericarditis, defined as inflammation of the pericardial sac surrounding the heart, is a clinically important condition with diverse causes [1, 2] and variable outcomes [2]. Although most patients recover with timely treatment, a subset may experience recurrent episodes or develop constrictive pericarditis, posing risks of morbidity and mortality [3, 4]. Identifying prognostic indicators to stratify risk and guide management remains an unmet clinical need [4].

Standard inflammatory biomarkers such as C-reactive protein (CRP) and erythrocyte sedimentation rate (ESR) are routinely used in the diagnosis and monitoring of pericarditis [2, 5, 6]. However, these markers may not capture the complex interplay among systemic inflammation, nutritional status, and hepatic synthetic function inherent in severe inflammatory states [7]. In cardiovascular research, composite biomarkers that integrate inflammatory and/ or nutritional components, such as the neutrophil-lymphocyte ratio [8], have gained increasing attention for their prognostic value in various cardiovascular conditions.

The CRP-to-albumin ratio (CAR) has emerged as a promising composite biomarker reflecting both inflammatory burden and nutritional reserve [9]. CRP rises rapidly as an acute-phase reactant in response to inflammatory stimuli, whereas serum albumin, a negative acute-phase protein, decreases during inflammation or catabolic states [7]. By combining both, CAR may provide a more comprehensive measure of the patient’s pathophysiological state [10, 11]. Recent pericarditis biomarker research emphasizes IL-1β and IL-6 signaling as key drivers of disease activity and recurrence [12–14]. Cytokine-induced acute-phase responses increase CRP while suppressing albumin synthesis, highlighting the importance of composite indices that reflect inflammatory burden and systemic reserve [12, 15]. In this context, CAR may provide a pragmatic admission marker of inflammatory–reserve disequilibrium in hospitalized pericarditis [12, 13].

Recent investigations have demonstrated the prognostic utility of CAR across multiple cardiovascular settings. Elevated CAR levels have been independently associated with long-term mortality in patients with acute coronary syndromes [16] and with increased long-term mortality among patients with chronic heart failure [17], demonstrating prognostic value beyond conventional inflammatory markers such as CRP or ESR. In large community-based cohorts such as the United Kingdom Biobank, higher CAR levels were also linked to incident cardiovascular disease and all-cause mortality [18]. Collectively, these findings support CAR as an integrative biomarker of systemic inflammation, metabolic stress, and nutritional status.

Despite growing evidence supporting CAR in diverse cardiovascular conditions, data on its prognostic value in hospitalized pericarditis, particularly for long-term outcomes, remain limited. To our knowledge, no large-scale real-world study has evaluated the association between baseline CAR and long-term all-cause mortality in this population [19]. Given the expanding therapeutic landscape and increasing emphasis on individualized management, reliable prognostic markers are urgently needed to guide risk stratification and patient follow-up in pericarditis [2, 20].

Accordingly, this study aimed to evaluate the association between baseline CAR and long-term all-cause mortality among patients hospitalized for pericarditis at a tertiary centre from Hong Kong using a subset of a prior study by our team [21]. We further explored the dose–response and threshold relationships using restricted cubic spline and segmented Cox regression models, and conducted prespecified subgroup analyses by age, sex, hypertension, and malignancy status, to evaluate the consistency of associations across clinically relevant subgroups.

## Methods

### Study design and population

This study used a subset of a previously conducted retrospective cohort study of adult patients hospitalized for pericarditis between January 1st, 2005 to December 31st, 2019 from a single tertiary centre [21]. The requirement for informed consent was specifically waived because of the retrospective design and use of deidentified data. The study was part of wider investigations approved by the Chinese University of Hong Kong–New Territories East Cluster Clinical Research Ethics Committee (https://www.crec.cuhk.edu.hk/approved-study-database/?dbr=&dbt=&dbn=tse+gary) on the use of ECGs for risk stratification (Approval numbers: 2018.309, 2018.643, 2019.338, 2019.361, and 2019.422), which contributed to the Doctor of Medicine degree of Dr. Gary Tse (https://www.proquest.com/docview/3252768154/278E578A36174385PQ). A letter from the MD Subcommittee of the Graduate School, The Chinese University of Hong Kong, authorizing the use of these data was provided. The associated datasets are publicly available via ProQuest (https://www.proquest.com/docview/3252768154/278E578A36174385PQ/2?%20Theses&sourcetype=Dissertations%20). The clinical database has been validated and widely applied in epidemiologic and cardiovascular outcomes research for single centre [22, 23] as well as multi-centre studies [24], including cross-cluster studies from the territory [25].

We identified all adult patients (aged ≥ 18 years) who were hospitalized with a primary diagnosis of pericarditis between January 1st, 2005, and December 31st, 2019 from a single tertiary centre. Pericarditis was clinically diagnosed during hospitalization according to the European Society of Cardiology (ESC) criteria, and ICD-10 discharge codes were used to identify eligible cases in CDARS for research. For patients with multiple hospitalizations, only the index admission was included for analysis. Patients were excluded if they had missing baseline measurements of CRP or serum albumin, or lacked survival outcome data.

### Data collection

Baseline demographic characteristics, comorbidities and laboratory parameters were extracted from the electronic health record system. Comorbid conditions—including hypertension, diabetes mellitus, sudden cardiac death (SCD), ischemic heart disease (IHD), acute myocardial infarction (AMI), malignant arrhythmia, peripheral vascular disease (PVD), malignancy, stroke or transient ischemic attack (TIA), chronic kidney disease (CKD), and chronic obstructive pulmonary disease (COPD)—were identified using ICD-10 diagnostic codes. The Charlson Comorbidity Index (CCI) was calculated for each patient to quantify baseline disease burden. Laboratory measurements obtained during the index hospitalization included CRP, serum albumin, complete blood count, renal function tests, serum electrolytes, lipid profile, prothrombin time (PT), activated partial thromboplastin time (APTT), and high-sensitivity troponin I (hs-TnI).

The CRP–to–albumin ratio (CAR) was calculated as CRP (mg/L) divided by serum albumin (g/L), and patients were categorized into quartiles according to its distribution in the cohort.

### Outcomes

The primary outcome was all-cause mortality. Mortality was ascertained through the Hong Kong Death Registry, ensuring complete mortality follow-up. Follow-up was calculated from index admission to death or administrative censoring.

### Statistical analysis

Continuous variables were summarized as means ± standard deviations for approximately normally distributed data or as medians with interquartile ranges for skewed data. Categorical variables were presented as counts (percentages). Group differences across CAR quartiles were evaluated using one-way ANOVA or the Kruskal–Wallis test for continuous variables and the chi-square test for categorical variables, as appropriate.

Given the right-skewed distribution of CAR and the presence of high-end outliers, we presented distribution plots and performed sensitivity analyses using 1st–99th percentile winsorization. Cox models were refitted using both raw and winsorized CAR to evaluate robustness.

To assess potential selection bias due to missing biomarkers, baseline characteristics of included versus excluded patients were compared using Student’s t test or Wilcoxon rank-sum test for continuous variables and the chi-square or Fisher’s exact test for categorical variables, as appropriate.

Survival probabilities were estimated using the Kaplan–Meier method, and differences across CAR quartiles were compared with the log-rank test. The association between CAR and all-cause mortality was examined using Cox proportional hazards regression. Hazard ratios (HRs) and 95% confidence intervals (CIs) were estimated for each quartile, with the lowest quartile serving as the reference group. P for trend was derived by treating quartiles as an ordinal variable. The proportional hazards assumption was assessed using Schoenfeld residuals.

To account for potential confounding, four sequential models were constructed: Model 1, unadjusted; Model 2, adjusted for age and sex; Model 3, additionally adjusted for comorbidities including SCD, IHD, AMI, malignant arrhythmia, hypertension, diabetes mellitus, PVD, malignancy, CKD, stroke/TIA, and COPD; and Model 4, further adjusted for clinically imbalanced baseline variables including heart rate, serum sodium, total cholesterol, triglycerides, LDL-C, and PT. To test the robustness of CAR modeling, we additionally examined alternative specifications, including dichotomized comparisons (Q1 vs. Q2–Q4 and CAR < 0.33 vs. ≥ 0.33) and a log-transformed model [log(CAR + 0.01)] in the same sequential adjustment framework.

To evaluate nonlinear and potential threshold associations, restricted cubic splines were fitted within the Cox model using the median CAR as the reference. Sensitivity analyses using 3 and 5 knots showed similar overall trends but greater instability at the extremes; thus, the 4-knot model, with knots placed at the 5th, 35th, 65th, and 95th percentiles, was retained for the main analysis. Rug plots were provided to visualize data density. Sensitivity spline analyses were repeated under less-adjusted models to verify consistency of curve patterns. The inflection point derived from the multivariable RCS curve was further examined using segmented Cox regression, and the likelihood-ratio test was applied to compare linear versus piecewise model fits. Given limited events in the lowest CAR range, threshold analyses were considered exploratory.

Prespecified subgroup analyses were performed according to age (< 65 vs. ≥ 65 years), sex, hypertension, and malignancy status. These subgroups were selected based on prior literature and biological plausibility, given their potential to modify the relationship between systemic inflammation, nutritional status, and mortality [26–28]. Interaction terms between CAR and each subgroup variable were included in the fully adjusted model to assess effect modification.

All statistical analyses were conducted using R software (version 4.3.1; R Foundation for Statistical Computing, Vienna, Austria). A two-sided p value < 0.05 was considered statistically significant.

## Results

### Baseline characteristics

**Fig. 1:**
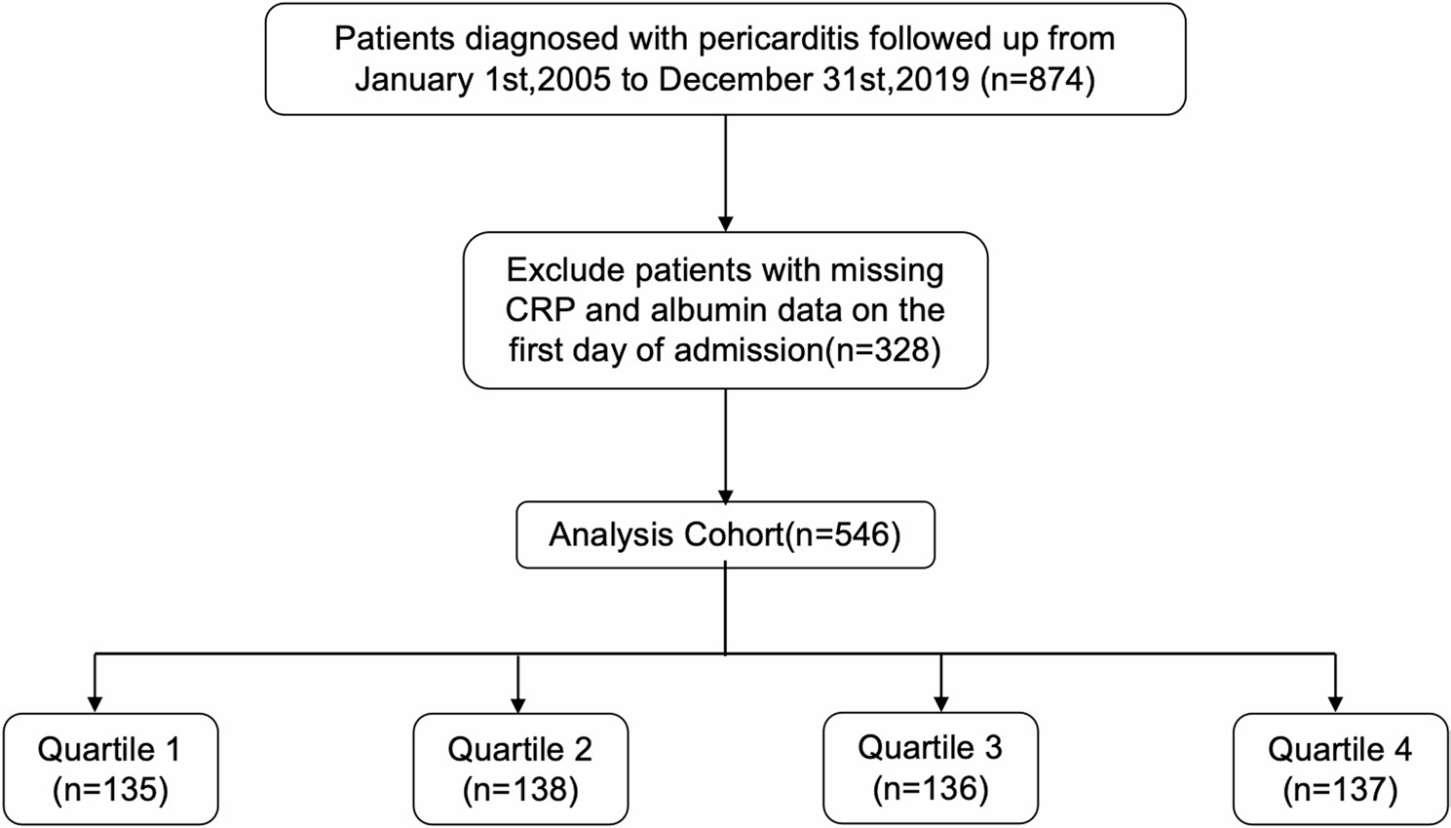
Flowchart of the study cohort. Flowchart illustrating patient inclusion and exclusion in the pericarditis cohort derived from the Clinical Data Analysis and Reporting System (CDARS).Abbreviations: CAR indicates C-reactive protein–to–albumin ratio; CDARS, Clinical Data Analysis and Reporting System

A total of 546 patients hospitalized for pericarditis were included and stratified into quartiles of the CAR: Q1 (0.01–0.17), Q2 (0.17–0.95), Q3 (0.95–2.72), and Q4 (2.72–27.27) (Fig. 1). Baseline characteristics of included versus excluded patients due to missing CRP or albumin measurements are presented in Supplementary Table S1. CAR exhibited a right-skewed distribution with a long upper tail; therefore, we provided distribution plots and performed sensitivity analyses using 1st–99th percentile winsorization. Refitted Cox models and raw versus winsorized spline curves yielded consistent overall patterns, suggesting limited influence of extreme values (Supplementary Figures S1–S3; Supplementary Tables S2–S3). The baseline characteristics across CAR quartiles are summarized in Table 1. Patients with higher CAR levels exhibited an overall worse clinical and biochemical profile. Specifically, elevated CAR was associated with higher heart rate (*P* < 0.001), lower serum sodium levels (*P* < 0.001), and decreased lipid concentrations—including total cholesterol, triglycerides, and low-density lipoprotein cholesterol (all *P* ≤ 0.022). Prothrombin time also increased progressively across CAR quartiles (*P* < 0.001). In addition, the proportion of male patients was higher in Q4 than Q1 (62.8% vs. 44.4%; *P* = 0.046).

**Table 1:** Baseline characteristics of patients with pericarditis according to quartiles of the C-Reactive Protein–to–Albumin ratio

Categories	Overall(*N* = 546)	Q1 (*N* = 135)	Q2 (*N* = 138)	Q3 (*N* = 136)	Q4 (*N* = 137)	*P*-value
Age, years	58.05 ± 17.35	60.39 ± 17.50	56.13 ± 19.34	57.69 ± 17.25	58.04 ± 14.97	0.242
Gender						0.046
Female	238 (43.59)	60 (44.44)	73 (52.90)	54 (39.71)	51 (37.23)	
Male	308 (56.41)	75 (55.56)	65 (47.10)	82 (60.29)	86 (62.77)	
Heart rate, (beats/min)	83.58 ± 21.70	74.42 ± 15.21	85.22 ± 21.97	87.96 ± 23.17	86.59 ± 22.92	< 0.001
Charlson	2.55 ± 2.33	2.32 ± 1.59	2.77 ± 2.66	2.60 ± 2.47	2.49 ± 2.44	0.440
Comorbidities						
SCD, n(%)	8 (1.47)	2 (1.48)	2 (1.45)	1 (0.74)	3 (2.19)	0.881
IHD, n(%)	45 (8.24)	14 (10.37)	13 (9.42)	7 (5.15)	11 (8.03)	0.425
AMI, n(%)	7 (1.28)	2 (1.48)	1 (0.72)	1 (0.74)	3 (2.19)	0.686
Malignant arrhythmia, n(%)	8 (1.47)	3 (2.22)	4 (2.90)	0 (0.00)	1 (0.73)	0.158
Hypertension, n(%)	90 (16.48)	28 (20.74)	22 (15.94)	25 (18.38)	15 (10.95)	0.157
DM, n(%)	19 (3.48)	4 (2.96)	8 (5.80)	1 (0.74)	6 (4.38)	0.096
PVD, n(%)	7 (1.28)	2 (1.48)	2 (1.45)	2 (1.47)	1 (0.73)	0.921
Malignancy, n(%)	74 (13.55)	15 (11.11)	17 (12.32)	24 (17.65)	18 (13.14)	0.418
CKD, n(%)	26 (4.76)	5 (3.70)	5 (3.62)	8 (5.88)	8 (5.84)	0.693
Stroke/TIA, n(%)	17 (3.11)	3 (2.22)	7 (5.07)	6 (4.41)	1 (0.73)	0.123
COPD, n(%)	5 (0.92)	1 (0.74)	2 (1.45)	1 (0.74)	1 (0.73)	1.000
Laboratory parameters						
WBC, ×10⁹/L	7.75 ± 3.76	7.42 ± 3.07	7.13 ± 3.49	8.20 ± 4.13	8.35 ± 4.21	0.083
RBC, ×10^12^/L	4.07 ± 0.83	4.23 ± 0.78	4.00 ± 0.72	3.95 ± 0.95	4.08 ± 0.85	0.126
Platelet, ×10⁹/L	224.41 ± 98.51	222.12 ± 90.52	227.89 ± 96.14	223.33 ± 109.94	224.24 ± 99.24	0.982
MCH, pg	30.20 ± 3.41	30.29 ± 3.43	29.98 ± 3.25	30.54 ± 3.67	29.98 ± 3.34	0.657
Scr, umol/L	84.00 (66.00, 115.00)	88.00 (73.00,111.00)	79.00 (63.25,107.50)	87.50 (65.75,119.25)	80.00 (63.00,124.00)	0.160
Total cholesterol, mmol/L	3.94 ± 1.37	4.71 ± 1.53	3.68 ± 1.14	4.06 ± 1.23	3.39 ± 1.28	< 0.001
Triglycerides, mmol/L	1.01 (0.76, 1.43)	1.05 (0.85,1.69)	0.85 (0.70,1.24)	1.05 (0.90,1.74)	1.08 (0.79,1.61)	0.022
LDL-C, mmol/L	2.32 ± 1.07	2.85 ± 1.26	2.12 ± 0.98	2.37 ± 1.07	1.98 ± 0.74	0.006
K⁺, mmol/L	4.08 ± 0.59	4.12 ± 0.56	4.02 ± 0.56	4.04 ± 0.62	4.16 ± 0.62	0.164
Na⁺, mmol/L	137.76 ± 4.55	139.40 ± 3.68	138.21 ± 4.20	137.31 ± 4.53	136.13 ± 5.07	< 0.001
hs-TnI, ng/L	0.02 (0.01, 0.08)	0.02 (0.01,0.06)	0.02 (0.01,0.07)	0.02 (0.01,0.06)	0.04 (0.01,0.24)	0.183
APPT, second	33.20 (30.30, 37.00)	33.15 (29.95,36.42)	33.30 (30.40,36.60)	33.20 (30.05,37.50)	33.30 (30.75,37.20)	0.863
PT, second	12.00 (10.80, 13.70)	11.00 (10.40,12.00)	11.80 (10.70,13.60)	12.60 (11.30,14.35)	13.25 (11.43,14.70)	< 0.001

Data are expressed as mean ± SD, median (IQR), or number (%). Group differences were assessed using one-way ANOVA, Kruskal–Wallis, or χ² tests, as appropriate

*AMI* indicates acute myocardial infarction, *APTT* Activated partial thromboplastin time, *CAR* C-reactive protein–to–albumin ratio, *CKD *Chronic kidney disease,* COPD* Chronic obstructive pulmonary disease, *DM* Diabetes mellitus, *hs-TnI* High-sensitivity troponin I, *IHD* Ischemic heart disease, *IQR* Interquartile range, *LDL-C* Low-density lipoprotein cholesterol, *MCH* Mean corpuscular hemoglobin, *PT* Prothrombin time, *PVD* Peripheral vascular disease,* RBC* Red blood cell,* SCD* Sudden cardiac death, *Scr* Serum creatinine, *SD* Standard deviation, *TIA* Transient ischemic attack, *WBC* White blood cell

### Kaplan–Meier survival analysis

**Fig. 2:**
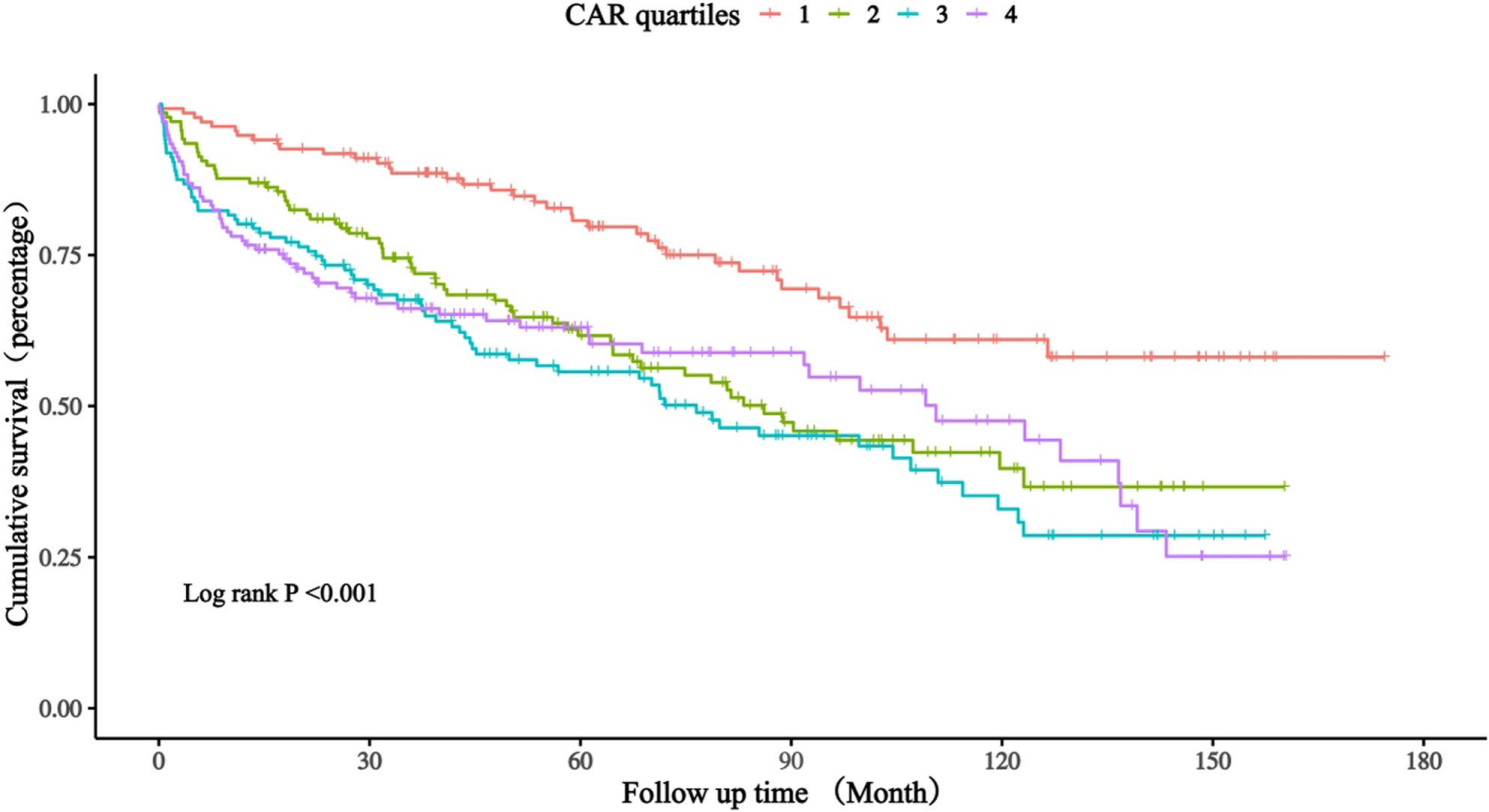
Kaplan–Meier Survival Curves for All-Cause Mortality According to Quartiles of the C-Reactive Protein–to–Albumin Ratio. Cumulative all-cause mortality curves across quartiles of the C-reactive protein–to–albumin ratio (Q1: 0.01–0.17; Q2: 0.17–0.95; Q3: 0.95–2.72; Q4: 2.72–27.27). Patients in higher quartiles showed progressively poorer survival (log-rank P < 0.001). Follow-up was measured from index admission to death or administrative censoring.Abbreviations: CAR indicates C-reactive protein–to–albumin ratio

During a median follow-up of 64 months, 239 deaths (43.8%) occurred. No patients were lost to follow-up for mortality ascertainment due to registry linkage. Kaplan–Meier survival curves revealed a graded, stepwise increase in all-cause mortality across CAR quartiles (log-rank *P* < 0.001) (Fig. 2). Patients in the upper quartiles (Q3–Q4) showed markedly higher cumulative mortality, with early and sustained divergence of survival curves throughout follow-up.

### Multivariable Cox regression analysis

Multivariable Cox proportional hazards regression was performed to identify significant predictors (Table 2). The proportional hazards assumption was assessed using Schoenfeld residuals, and no evidence of violation was observed for CAR in the fully adjusted model (Supplementary Table S4). In the fully adjusted model (Model 4), CAR remained independently associated with all-cause mortality. When modeled as a continuous variable, each unit increase in CAR was associated with a 5% higher risk of death (HR, 1.05; 95% CI, 1.01–1.10; *P* = 0.031). When analyzed as quartiles, using the lowest quartile (Q1) as the reference, higher CAR categories were consistently associated with increased mortality risk. Adjusted HRs were 2.04 (95% CI, 1.34–3.09; *P* < 0.001) for Q2, 2.21 (95% CI, 1.47–3.32; *P* < 0.001) for Q3, and 1.93 (95% CI, 1.26–2.95; *P* = 0.002) for Q4 (P for trend = 0.004). These associations remained consistent across Models 1–4; notably, Model 4 additionally adjusted for heart rate, serum sodium, lipid profile, and prothrombin time, indicating that the observed relationship was not driven by demographic factors, comorbidities, or these baseline imbalances. Additional analyses using dichotomized and log-transformed specifications of CAR yielded consistent associations with mortality across sequential models (Supplementary Table S5).

**Table 2:** Cox proportional hazard ratios for All-Cause mortality according to quartiles and continuous values of the C-Reactive Protein–to–Albumin ratio

Categories	Model 1				Model 2				Model 3			Model 4		
	HR(95% CI)	*P*-value	*P* for trend		HR(95% CI)	*P*-value	*P* for trend		HR (95% CI)	*P*-value	*P* for trend	HR (95% CI)	*P*-value	*P* for trend
Continuous variable per 1 unit	1.04 (1.01 ~ 1.09)	0.048			1.05 (1.01 ~ 1.10)	0.018			1.05 (1.01 ~ 1.10)	0.016		1.05 (1.01 ~ 1.10)	0.031	
Quartile^a^			< 0.001				< 0.001				< 0.001			0.004
Q1 (*N* = 135)	Ref.				Ref.				Ref.			Ref.		
Q2 (*N* = 138)	2.03 (1.36 ~ 3.04)	< 0.001			2.17 (1.45 ~ 3.25)	< 0.001			2.31 (1.53 ~ 3.50)	< 0.001		2.04 (1.34 ~ 3.09)	< 0.001	
Q3 (*N* = 136)	2.48 (1.68 ~ 3.67)	< 0.001			2.69 (1.81 ~ 3.99)	< 0.001			2.65 (1.78 ~ 3.94)	< 0.001		2.21 (1.47 ~ 3.32)	< 0.001	
Q4 (*N* = 137)	2.18 (1.45 ~ 3.26)	< 0.001			2.30 (1.54 ~ 3.46)	< 0.001			2.39 (1.58 ~ 3.60)	< 0.001		1.93 (1.26 ~ 2.95)	0.002	

Hazard ratios (HRs) and 95% confidence intervals (CIs) were derived from Cox proportional hazards models across four sequential models of adjustment. The lowest quartile served as the reference group

Model 1: unadjusted

Model 2: adjusted for age and sex

Model 3: adjusted for age, sex, and comorbidities including sudden cardiac death (SCD), ischemic heart disease (IHD), acute myocardial infarction (AMI), malignant arrhythmia, hypertension, diabetes mellitus, peripheral vascular disease (PVD), malignancy, chronic kidney disease (CKD), stroke or transient ischemic attack (TIA), and chronic obstructive pulmonary disease (COPD)

Model 4: adjusted for age, sex, comorbidities and heart rate, serum sodium, total cholesterol, triglycerides, low-density lipoprotein cholesterol (LDL-C), and prothrombin time (PT)

^a^CAR quartiles were defined as Q1 (0.01–0.17), Q2 (0.17–0.95), Q3 (0.95–2.72), and Q4 (2.72–27.27)

*CAR* indicates C-reactive protein–to–albumin ratio, *HR* Hazard ratio, *CI* Confidence interval, *SCD* Sudden cardiac death, *IHD* Ischemic heart disease, *AMI* Acute myocardial infarction, *PVD *Peripheral vascular disease, *CKD* Chronic kidney disease, *TIA* Transient ischemic attack, COPD Chronic obstructive pulmonary disease

### Dose–Response relationship

**Fig. 3:**
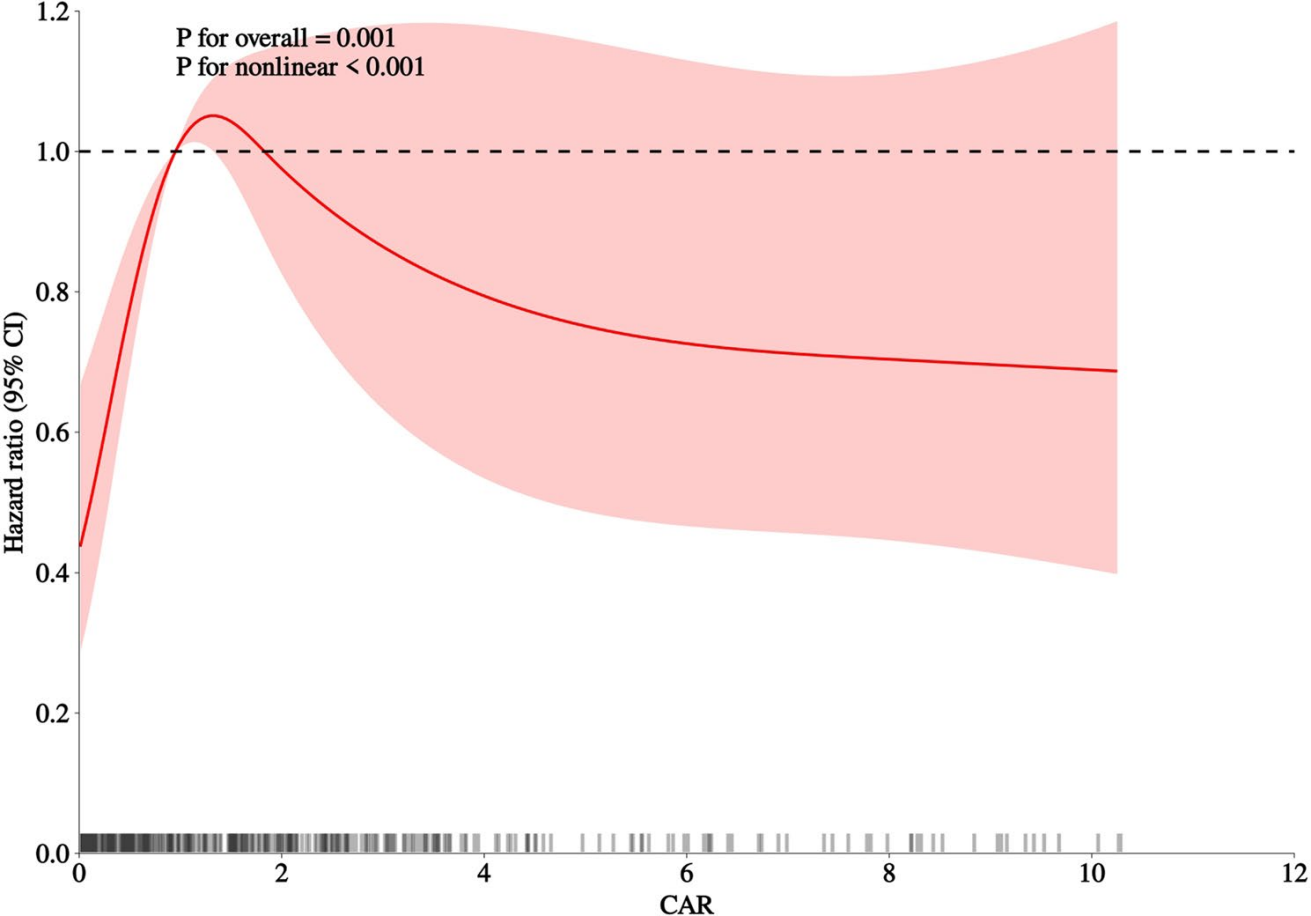
Multivariable-Adjusted Restricted Cubic Spline Analysis of the Association Between the C-Reactive Protein–to–Albumin Ratio and All-Cause Mortality. Restricted cubic spline analysis illustrating the nonlinear association between the C-reactive protein–to–albumin ratio (CAR) and all-cause mortality. Models were adjusted for age, sex, comorbidities and heart rate, serum sodium, total cholesterol, triglycerides, low-density lipoprotein cholesterol (LDL-C), and prothrombin time (PT) as in Model 4. The solid line represents the adjusted hazard ratio, and the shaded area indicates the 95% confidence interval. Knots were placed at the 5th, 35h, 65h and 95th percentiles of CAR (P=0.001 for overall association; P＜0.001 for nonlinearity). Rug plots are shown along the x-axis to indicate data density. Abbreviations: CAR indicates C-reactive protein–to–albumin ratio; HR, hazard ratio; CI, confidence interval

**Fig. 4:**
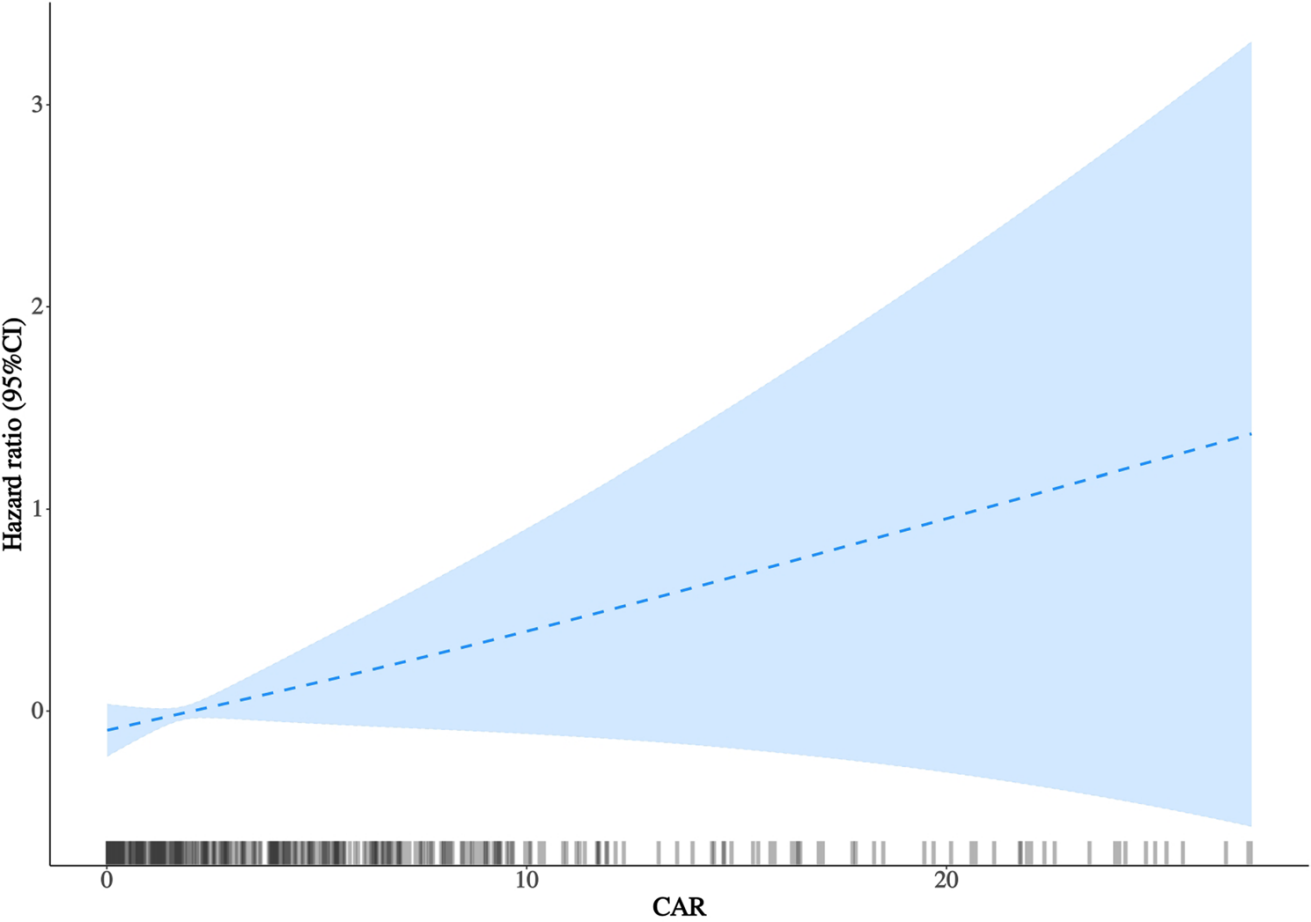
Segmented Cox Regression Illustrating the Threshold Effect of the C-Reactive Protein–to–Albumin Ratio on All-Cause Mortality. Segmented Cox regression illustrating the threshold effect of the C-reactive protein–to–albumin ratio (CAR) on all-cause mortality. The solid line represents the adjusted hazard ratio, and the shaded area indicates the 95% confidence interval. Models were adjusted as in Model 4, including age, sex, comorbidities, heart rate, serum sodium, total cholesterol, triglycerides, low-density lipoprotein cholesterol (LDL-C), and prothrombin time (PT). Below the threshold (CAR ≈ 0.33), mortality risk increased steeply with CAR, whereas above this value the association plateaued (likelihood-ratio P = 0.016). Rug plots are shown along the x-axis to indicate data density. Abbreviations: CAR indicates C-reactive protein–to–albumin ratio; HR, hazard ratio; CI, confidence interval

RCS analysis demonstrated a significant overall association between CAR and all-cause mortality (P overall = 0.001) and clear nonlinearity (P nonlinearity < 0.001) (Fig. 4). This pattern remained broadly consistent in sensitivity analyses using 3 and 5 knots (Supplementary Figure S6). The curve revealed a steep risk increase at lower CAR levels that gradually flattened at higher ranges. Rug plots in Figs. 4and 3 show that CAR values were sparsely distributed at the extremes.

To further evaluate threshold effects, segmented Cox regression was performed (Fig. 3), identifying an inflection point at a CAR value of approximately 0.33. Below this threshold, mortality risk increased sharply (adjusted HR: 24.67; 95% CI, 16.66–125.21; *P* = 0.010), although the wide confidence intervals reflect limited events in this range. Above this level, the association plateaued (adjusted HR: 1.00; 95% CI, 0.94–1.06; *P* = 0.945). The piecewise model provided a significantly better fit than a single linear term (likelihood-ratio *P* = 0.016) (Supplementary Table S6).

### Sensitivity and subgroup analyses

Sensitivity analyses demonstrated that the association between CAR and all-cause mortality remained consistent across alternative model specifications. Restricted cubic spline analyses based on unadjusted, age- and sex-adjusted, and age-, sex- and comorbidities-adjusted models (Supplementary Figure S5) revealed similar dose–response patterns, confirming the robustness of the observed nonlinear relationship.

**Fig. 5:**
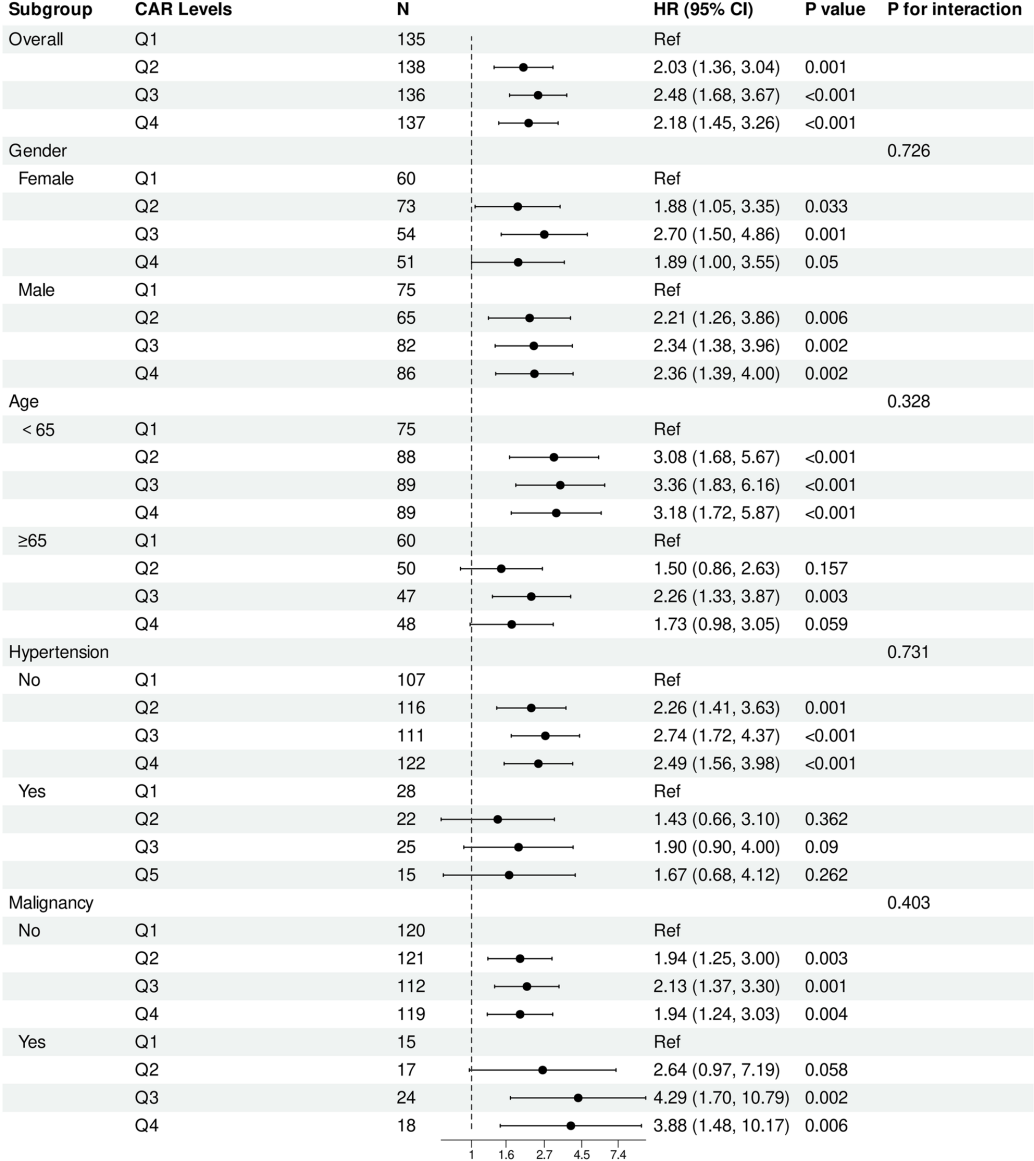
Subgroup Analysis of the Association Between the C-Reactive Protein–to–Albumin Ratio and All-Cause Mortality. Forest plot showing hazard ratios (HRs) and 95% confidence intervals (CIs) for all-cause mortality across quartiles of the C-reactive protein–to–albumin ratio (CAR) in prespecified subgroups defined by age, sex, hypertension, and malignancy status.Multivariable Cox regression models were used to estimate hazard ratios within each subgroup, adjusting for demographic and clinical covariates. Q1 served as the reference quartile. P-interaction values indicate effect modification across subgroups. Wider CIs in certain subgroups reflect limited sample size and event counts.Abbreviations: CAR indicates C-reactive protein–to–albumin ratio; HR, hazard ratio; CI, confidence interval

In prespecified subgroup analyses (Fig. 5 and Table S7), the association between higher CAR quartiles and all-cause mortality was generally directionally similar across subgroups defined by sex, age, hypertension, and malignancy status. No significant interactions were observed (all P for interaction > 0.05). However, several subgroup estimates showed wide confidence intervals, particularly in smaller strata such as malignancy, reflecting limited event counts.

## Discussion

### Principal findings

In this cohort of patients hospitalized for pericarditis, CAR emerged as an independent predictor of long-term all-cause mortality. This association remained robust after multivariable adjustment for demographic and clinical covariates, including additional models that accounted for key baseline imbalances, highlighting the incremental prognostic utility of CAR beyond conventional clinical and inflammatory markers.

Restricted cubic spline and segmented Cox analyses revealed a nonlinear dose–response relationship, characterized by a steep rise in mortality risk at lower CAR values followed by a plateau at higher ranges, with a potential threshold at a CAR value of approximately 0.33. Given the limited event density at very low CAR values and the resulting wide confidence intervals, the low-range threshold signal around CAR ≈ 0.33 should be interpreted as exploratory. This overall nonlinear pattern remained directionally consistent in sensitivity analyses using alternative knot specifications and approaches to mitigate the influence of extreme values, and the overall nonlinear pattern may suggest a potential low-range inflection that warrants external validation.

These associations were generally directionally similar across prespecified subgroups (age, sex, hypertension, and malignancy status) and persisted in sensitivity analyses using alternative adjustment models, as well as alternative CAR specifications, supporting the stability and robustness of the findings, although precision was limited in smaller strata with wide confidence intervals.

### Comparison with previous studies

Our findings are consistent with prior evidence underscoring the prognostic significance of CAR across a broad spectrum of cardiovascular disorders. In patients with acute coronary syndromes, elevated CAR has been independently associated with higher long-term mortality and adverse cardiac events [16]. Among coronary populations undergoing percutaneous coronary intervention, incorporation of CAR into predictive models further improved the discrimination of all-cause and cardiovascular mortality [29].

Besides IHD, CAR has demonstrated prognostic relevance in other critical cardiovascular settings. Higher CAR levels were independently related to both in-hospital and six-month mortality in survivors of out-of-hospital cardiac arrest [30]. In heart-failure populations, elevated CAR correlated with worse functional profiles—such as higher New York Heart Association (NYHA) class—and was associated with increased in-hospital and out-of-hospital mortality [31]. In cohorts with heart failure and reduced ejection fraction, CAR independently predicted long-term mortality [32].

Collectively, these studies identify CAR as a robust, integrative biomarker reflecting systemic inflammation and nutritional reserve—two interrelated processes central to cardiovascular pathophysiology. However, its prognostic utility may vary by clinical context. In a large community-based analysis from the UK Biobank, CAR did not outperform CRP in predicting incident cardiovascular events or mortality among otherwise healthy individuals, suggesting that its predictive value may be more pronounced in populations characterized by active inflammation or metabolic stress [18].

A small study in related pericardial settings has explored CAR indices for short-term outcomes [33], but evidence in hospitalized pericarditis with long-term follow-up remains limited. Our study extends this body of evidence by demonstrating a nonlinear association between CAR and mortality in pericarditis, with an inflection point near CAR = 0.33, below which mortality risk increased steeply before reaching a plateau. To our knowledge, few prior studies have examined such nonlinear or threshold effects in CAR-outcome relationships, highlighting the potential clinical relevance of our findings in this population.

### Pathophysiological mechanisms

The prognostic relevance of CAR in pericarditis likely reflects the integrated effects of systemic inflammation, hepatic synthetic function, and nutritional reserve. Elevated CRP levels signify activation of innate immune pathways and the intensity of systemic inflammation [34, 35]. Proinflammatory cytokines such as interleukin-1β, interleukin-6, and tumor necrosis factor-α stimulate hepatic CRP synthesis through NF-κB–dependent signaling. This cytokine-driven activation amplifies downstream inflammatory cascades, promoting pericardial injury, effusion, and fibrosis [36, 37]. Consistent with prior evidence, higher CRP levels have been associated with disease recurrence and an increased risk of constrictive remodeling in pericarditis [38, 39].

Conversely, hypoalbuminemia arises from cytokine-mediated suppression of hepatic synthesis [40], increased vascular permeability [40], and catabolic nutritional imbalance during acute systemic stress [41]. Low albumin not only indicates impaired nutritional and synthetic capacity but also contributes to oxidative stress and reduced plasma oncotic pressure [41], both of which can exacerbate myocardial and pericardial inflammation [42]. Thus, an elevated CAR simultaneously captures heightened inflammatory burden and diminished homeostatic reserve—two interdependent processes that jointly drive adverse cardiovascular outcomes.

Unlike atherosclerotic or myocardial diseases, pericardial inflammation is characterized by an exudative cytokine milieu and fibrinous reaction within the pericardial cavity [2]. In this setting, hypoalbuminemia may further aggravate effusive-constrictive physiology by reducing plasma oncotic pressure, promoting pericardial effusion, and perpetuating the inflammatory–fibrotic cycle [43, 44]. This potential mechanism may provide a biological rationale linking elevated CAR to worse clinical outcomes in pericarditis [45], although pericarditis-specific imaging markers were not captured in the present dataset.

The observed CAR threshold near 0.33 may represent a biologically meaningful transition point. At lower CAR levels, even modest inflammation coupled with early albumin decline may trigger endothelial dysfunction and immune-metabolic activation, producing a disproportionate rise in mortality risk. Alternatively, elevated risk at the lowest CAR range may reflect chronic immune exhaustion or severe malnutrition, where suppressed inflammatory responses coexist with impaired physiological reserve, leading to vulnerability despite low apparent inflammation [45–47]. Beyond this range, further CAR elevation—largely driven by rising CRP levels— may reflect persistent inflammation without additional deterioration of systemic homeostasis, resulting in a plateau effect. This curvilinear pattern supports the concept that early disruption of inflammatory-nutritional equilibrium, rather than extreme inflammation alone, underlies the excess risk observed in pericarditis. Further validation in independent cohorts is needed.

### Clinical implications

Given that our cohort comprised hospitalized patients in a tertiary centre, CAR-based risk stratification in this study is most applicable to real-world inpatient settings rather than lower-risk outpatient populations. Our findings support the clinical utility of CAR as a simple, inexpensive, and readily available biomarker for early risk stratification in pericarditis. Because it is derived from routinely measured laboratory parameters, CAR can be easily implemented across diverse clinical settings. In this study, a threshold around CAR ≈ 0.33 was observed as a potential risk-enrichment point for identifying patients at disproportionately higher risk, suggesting that even modest elevations may carry important prognostic implications.

Importantly, CAR provides an objective and quantitative assessment of systemic inflammation and nutritional reserve, complementing established clinical risk factors such as fever, large pericardial effusion, and poor response to nonsteroidal anti-inflammatory drugs. This framework aligns with risk-adapted management strategies endorsed by the European Society of Cardiology and the American Heart Association/ American College of Cardiology, although current guidelines have yet to incorporate biomarker-based risk tools [3, 48].

Beyond baseline measurement, serial CAR monitoring may assist in tracking inflammatory activity, evaluating therapeutic response, and informing treatment adjustments—an approach consistent with emerging evidence supporting dynamic biomarker-guided follow-up in recurrent pericarditis [4, 6]. In the longer term, individualized CAR-based risk profiles could enhance shared decision-making and improve communication between clinicians and patients regarding prognosis and care planning.

### Limitations

This study has several limitations. First, the retrospective observational design is inherently subject to residual confounding, despite comprehensive multivariable adjustment. Although major demographic characteristics, comorbidities, and baseline laboratory variables were accounted for, unmeasured factors such as nutritional indices, proinflammatory cytokines, medication adherence, and comorbidity severity could have influenced both CAR levels and outcomes [49]. In addition, key pericarditis-specific prognostic features—including effusion size, tamponade status, recurrence history, etiologies, and anti-inflammatory therapies (e.g., colchicine or corticosteroids)—were not available in this administrative dataset. Therefore, residual confounding by unmeasured disease severity cannot be fully excluded, and CAR may partly reflect pericarditis activity.

Second, patients without admission CRP or albumin were excluded, which may introduce selection bias. Although baseline characteristics between included and excluded patients were broadly comparable (Supplementary Table S1), nonrandom missingness cannot be fully excluded.

Third, CAR was measured only at baseline; temporal changes during hospitalization or follow-up were not captured. Thus, the prognostic significance of dynamic CAR trajectories remains unexplored. Previous evidence has suggested that dynamic inflammatory and nutritional responses may offer additional prognostic insight beyond baseline values [50].

Fourth, this analysis was conducted in Chinese patients from a single tertiary centre in Hong Kong, which may limit generalizability to populations with different ethnic backgrounds, healthcare systems, or pericarditis etiology distributions. In particular, infectious causes, including tuberculosis, may be relatively more prevalent in our setting, which could influence inflammatory profiles and the CAR–outcome relationship. The relatively high long-term mortality likely reflects the case-mix of a tertiary hospitalized population with substantial comorbidity burden and potentially more complex etiologies. External validation in geographically, ethnically, and etiologically diverse cohorts is warranted [51].

Finally, as with all retrospective studies using electronic records, diagnostic accuracy and event classification depend on data completeness and coding practices. Although segmented Cox regression suggested a possible low-range threshold around CAR ≈ 0.33, the number of patients and events in this range was limited, resulting in wide confidence intervals; therefore, this exploratory finding should be interpreted cautiously and validated in larger prospective multicenter studies.

## Conclusions

In this real-world cohort of patients hospitalized for pericarditis, the CAR was independently associated with long-term all-cause mortality. Both restricted cubic spline and segmented Cox regression analyses revealed a nonlinear relationship, with a threshold near CAR = 0.33, below which mortality risk increased steeply before reaching a plateau.

## Supplementary Information

Supplementary Material 1.

## Data Availability

This study used a subset of a previously conducted retrospective cohort of adult patients hospitalized for pericarditis from a single tertiary centre in Hong Kong (2005–2019). The requirement for informed consent was waived because all data were deidentified. The associated datasets supporting this analysis are available through ProQuest as part of the Doctor of Medicine thesis of Dr. Gary Tse (https://www.proquest.com/docview/3252768154/278E578A36174385PQ).Due to institutional policy and patient privacy regulations, the raw electronic health record data cannot be openly shared. Researchers who wish to access additional deidentified data may submit a reasonable request to the corresponding author and obtain approval from the relevant institutional review board.
