# Supplementary material for: C-reactive protein-to-albumin ratio as a novel prognostic biomarker for long-term mortality in pericarditis: a real-world study
Source: BMC Cardiovasc Disord. 2025 Dec 30;26:100. doi: 10.1186/s12872-025-05464-3 (PMC12866473; doi:10.1186/s12872-025-05464-3)
Supplement: Supplementary file 1 — Supplementary Material 1. [file 12872_2025_5464_MOESM1_ESM.docx]

**Supplementary Appendix**

**Supplementary Table S1.** Baseline characteristics of included versus excluded patients based on availability of C-reactive protein and albumin measurements.

| **Categories** | **Excluded (N= 328)** | **Included (N= 546)** | **P-value** |
| --- | --- | --- | --- |
| Age, years | 58.37 ± 17.02 | 58.05 ± 17.35 | 0.787 |
| Gender |  |  | 0.932 |
| Female | 142 (43.29) | 238 (43.59) |  |
| Male | 186 (56.71) | 308 (56.41) |  |
| Heart rate,(beats/min) | 83.00 ± 20.91 | 83.58 ± 21.70 | 0.700 |
| Charlson | 2.83 ± 2.61 | 2.55 ± 2.33 | 0.856 |
| **Comorbidities** |  |  |  |
| SCD, n(%) | 4 (1.21) | 8 (1.47) | 0.573 |
| IHD, n(%) | 28 (8.53) | 45 (8.24) | 0.437 |
| AMI, n(%) | 6 (1.83) | 7 (1.28) | 0.383 |
| Malignant arrhythmia, n(%) | 5 (1.52) | 8 (1.47) | 1.000 |
| Hypertension, n(%) | 65 (19.82) | 90 (16.48) | 0.212 |
| DM, n(%) | 6 (1.83) | 19 (3.48) | 0.156 |
| PVD, n(%) | 5 (1.52) | 7 (1.28) | 1.000 |
| Malignancy, n(%) | 45 (13.71) | 74 (13.55) | 0.395 |
| CKD, n(%) | 22 (6.71) | 26 (4.76) | 0.222 |
| Stroke/TIA, n(%) | 8 (2.44) | 17 (3.11) | 0.562 |
| COPD, n(%) | 3 (0.91) | 5 (0.92) | 1.000 |
| **Laboratory parameters** |  |  |  |
| WBC, ×10⁹/L | 8.04 ± 3.70 | 7.75 ± 3.76 | 0.390 |
| RBC, ×10^12^/L | 4.11 ± 0.74 | 4.07 ± 0.83 | 0.566 |
| Platelet，×10⁹/L | 238.03 ± 101.34 | 224.41 ± 98.51 | 0.129 |
| MCH, pg | 30.19 ± 3.10 | 30.20 ± 3.41 | 0.974 |
| Scr, umol/L | 81.00 (64.75, 103.25) | 84.00 (66.00, 115.00) | 0.238 |
| Total cholesterol, mmol/L | 4.08 ± 0.79 | 3.94 ± 1.37 | 0.052 |
| Triglycerides, mmol/L | 1.06 (0.69, 1.54) | 1.01 (0.76, 1.43) | 0.953 |
| LDL-C, mmol/L | 2.63 ± 0.60 | 2.32 ± 1.07 | 0.219 |
| K⁺, mmol/L | 4.08 ± 0.71 | 4.08 ± 0.59 | 0.940 |
| Na⁺, mmol/L | 137.93 ± 4.64 | 137.76 ± 4.55 | 0.702 |
| hs-TnI, ng/L | 1.30 (0.99, 1.58) | 1.03 (0.80, 1.40) | 0.051 |
| APPT, second | 33.30 (29.92, 35.65) | 33.20 (30.30, 37.00) | 0.093 |
| PT, second | 11.60 (10.70, 13.35) | 12.00 (10.80, 13.70) | 0.121 |

Group differences were assessed using Student’s t test, Wilcoxon rank-sum test, or χ²/Fisher’s exact test, as appropriate.

**Abbreviations:** AMI indicates acute myocardial infarction; APTT, activated partial thromboplastin time; CAR, C-reactive protein–to–albumin ratio; CKD, chronic kidney disease; COPD, chronic obstructive pulmonary disease; DM, diabetes mellitus; hs-TnI, high-sensitivity troponin I; IHD, ischemic heart disease; IQR, interquartile range; LDL-C, low-density lipoprotein cholesterol; MCH, mean corpuscular hemoglobin; PT, prothrombin time; PVD, peripheral vascular disease; RBC, red blood cell; SCD, sudden cardiac death; Scr, serum creatinine; SD, standard deviation; TIA, transient ischemic attack; WBC, white blood cell.

**Supplementary Table S2.** Distribution of raw and winsorized C-reactive protein-to-albumin ratio values.

| **Metric** | **Raw CAR** | **Winsorized CAR (1–99%)** |
| --- | --- | --- |
| N | 546 | 546 |
| Minimum | 0.01 | 0.01 |
| 1st percentile | 0.01 | 0.01 |
| Median | 0.95 | 0.95 |
| 99th percentile | 11.06 | 11.01 |
| Maximum | 27.27 | 11.06 |

This table summarizes the distribution of baseline CAR values in the study cohort using both raw and winsorized data. Winsorization was performed at the 1st–99th percentiles to reduce the influence of extreme high-end values; therefore, the maximum value in the winsorized column equals the 99th percentile of the raw CAR distribution. The table reports N, minimum, 1st percentile, median, 99th percentile, and maximum values for each specification.
**Abbreviations:** CAR indicates C-reactive protein–to–albumin ratio.

**Supplementary Figure S1.** Paired natural cubic spline analyses of raw and winsorized C-reactive protein-to-albumin ratio.

**
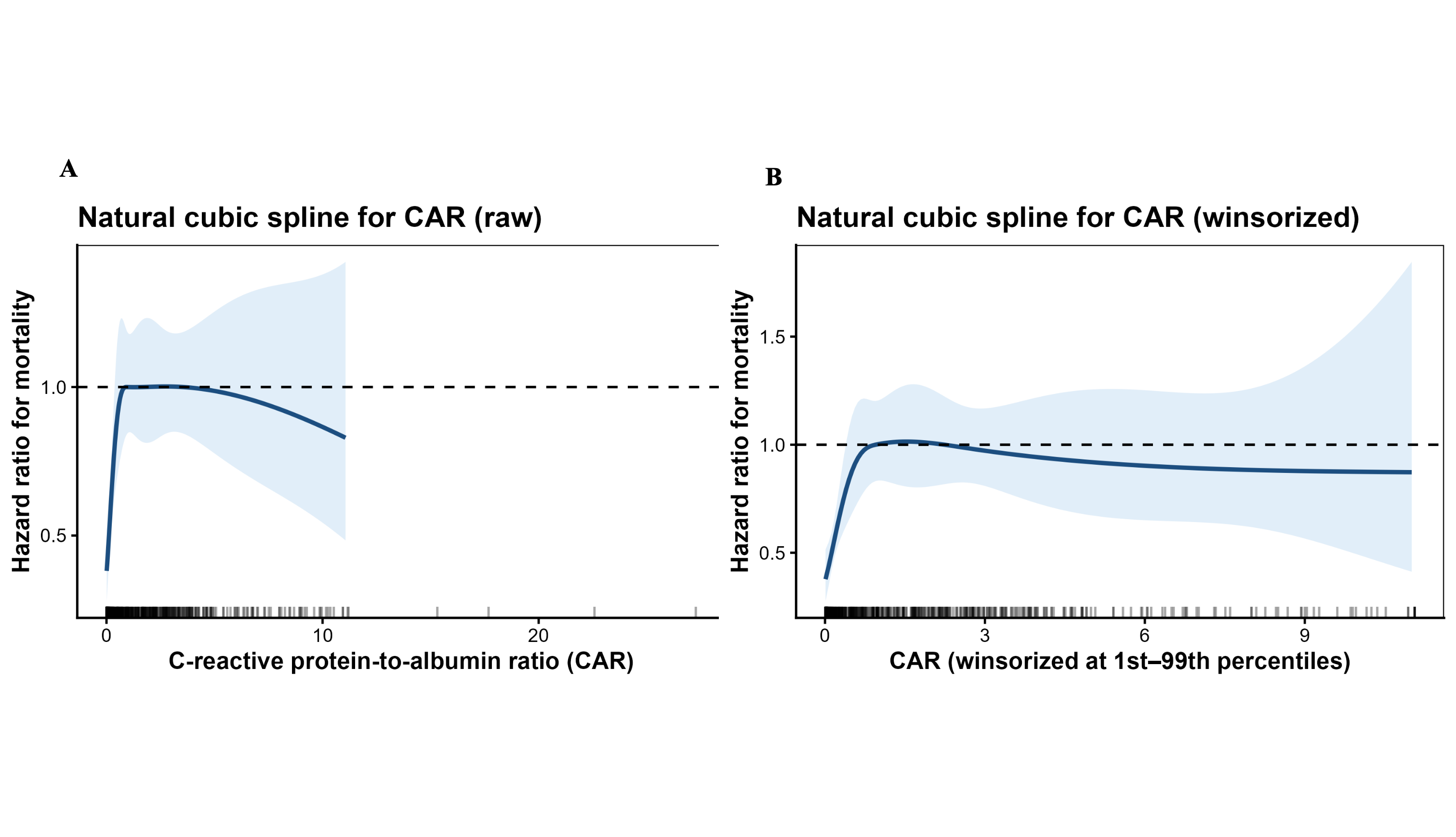
**

Panel A shows the natural cubic spline curve for baseline raw CAR, and Panel B shows the corresponding curve for CAR winsorized at the 1st–99th percentiles, presented to assess whether extreme high-end values materially influence the nonlinear pattern of the CAR–mortality association. The solid line represents the adjusted hazard ratio and the shaded area indicates the 95% confidence interval. Rug plots along the x-axis indicate the distribution and density of CAR values across the cohort.
**Abbreviations:** CAR indicates C-reactive protein–to–albumin ratio; HR, hazard ratio; CI, confidence interval.

**Supplementary Table S3.** Cox regression models comparing raw and winsorized C-reactive protein-to-albumin ratio.

| **Model** | **HR** | **95% CI** | **p-value** |
| --- | --- | --- | --- |
| Raw CAR | 1.04 | 1.00–1.09 | 0.048 |
| Winsorized CAR | 1.06 | 1.01–1.11 | 0.020 |

This table presents Cox proportional hazards models evaluating the association between baseline CAR and all-cause mortality using raw and 1st–99th percentile winsorized CAR values. The comparison was performed to assess whether extreme values materially influenced the estimated effect of CAR. Hazard ratios (HRs) and 95% confidence intervals (CIs) are reported for each specification.
**Abbreviations:** CAR indicates C-reactive protein–to–albumin ratio; HR, hazard ratio; CI, confidence interval.

**Supplementary Table S4.** Schoenfeld residual test for the proportional hazards assumption.

| **Variable** | **χ²** | **df** | **p-value** |
| --- | --- | --- | --- |
| CAR (winsorized 1–99%) | 0.74 | 1 | 0.391 |

This table summarizes the results of the Schoenfeld residual-based test evaluating the proportional hazards assumption for baseline CAR in the Cox model. The test was performed using the 1st–99th percentile winsorized CAR specification to ensure robustness against extreme values. The chi-square statistic (χ²), degrees of freedom (df), and corresponding p-value are presented. A non-significant p-value indicates no evidence of violation of the proportional hazards assumption for CAR.
**Abbreviations:** CAR indicates C-reactive protein–to–albumin ratio; χ², chi-square; df, degrees of freedom.

**Supplementary Table S5. Alternative specifications of C-reactive protein-to-albumin ratio for all-cause mortality.**

| **Specification** | **Model** | **HR (95% CI)** | **p-value** |
| --- | --- | --- | --- |
| Q1 vs Q2–Q4 | Model 1 | 0.44 (0.31–0.63) | <0.001 |
|  | Model 2 | 0.41 (0.29–0.58) | <0.001 |
|  | Model 3 | 0.40 (0.28–0.57) | <0.001 |
|  | Model 4 | 0.45 (0.22–0.52) | 0.021 |
| <0.33 vs ≥0.33 | Model 1 | 0.50 (0.37–0.67) | <0.001 |
|  | Model 2 | 0.48 (0.36–0.65) | <0.001 |
|  | Model 3 | 0.48 (0.35–0.65) | <0.001 |
|  | Model 4 | 0.42 (0.18–0.97) | 0.043 |
| log(CAR+0.01) | Model 1 | 1.19 (1.10–1.28) | <0.001 |
|  | Model 2 | 1.20 (1.12–1.30) | <0.001 |
|  | Model 3 | 1.21 (1.12–1.31) | <0.001 |
|  | Model 4 | 1.15 (1.09–1.37) | 0.025 |

This table presents Cox proportional hazards models evaluating the association between baseline CAR and all-cause mortality using dichotomized and log-transformed specifications to assess robustness of the primary findings. For dichotomized analyses, Q2–Q4 and CAR ≥ 0.33 were used as the reference groups, respectively; therefore, HRs < 1 for Q1 vs Q2–Q4 and CAR < 0.33 vs ≥ 0.33 indicate lower mortality risk in the lower-CAR group compared with the higher-CAR reference group. For the continuous alternative specification, log(CAR + 0.01) was modeled per 1-unit increase; thus, HRs > 1 indicate increased mortality risk with higher log-transformed CAR. Models 1–4 were constructed as described in the Methods.
**Abbreviations:** CAR indicates C-reactive protein–to–albumin ratio; HR, hazard ratio; CI, confidence interval.

**Supplementary Figure S2.** Distribution of the C-reactive protein-to-albumin ratio.

**
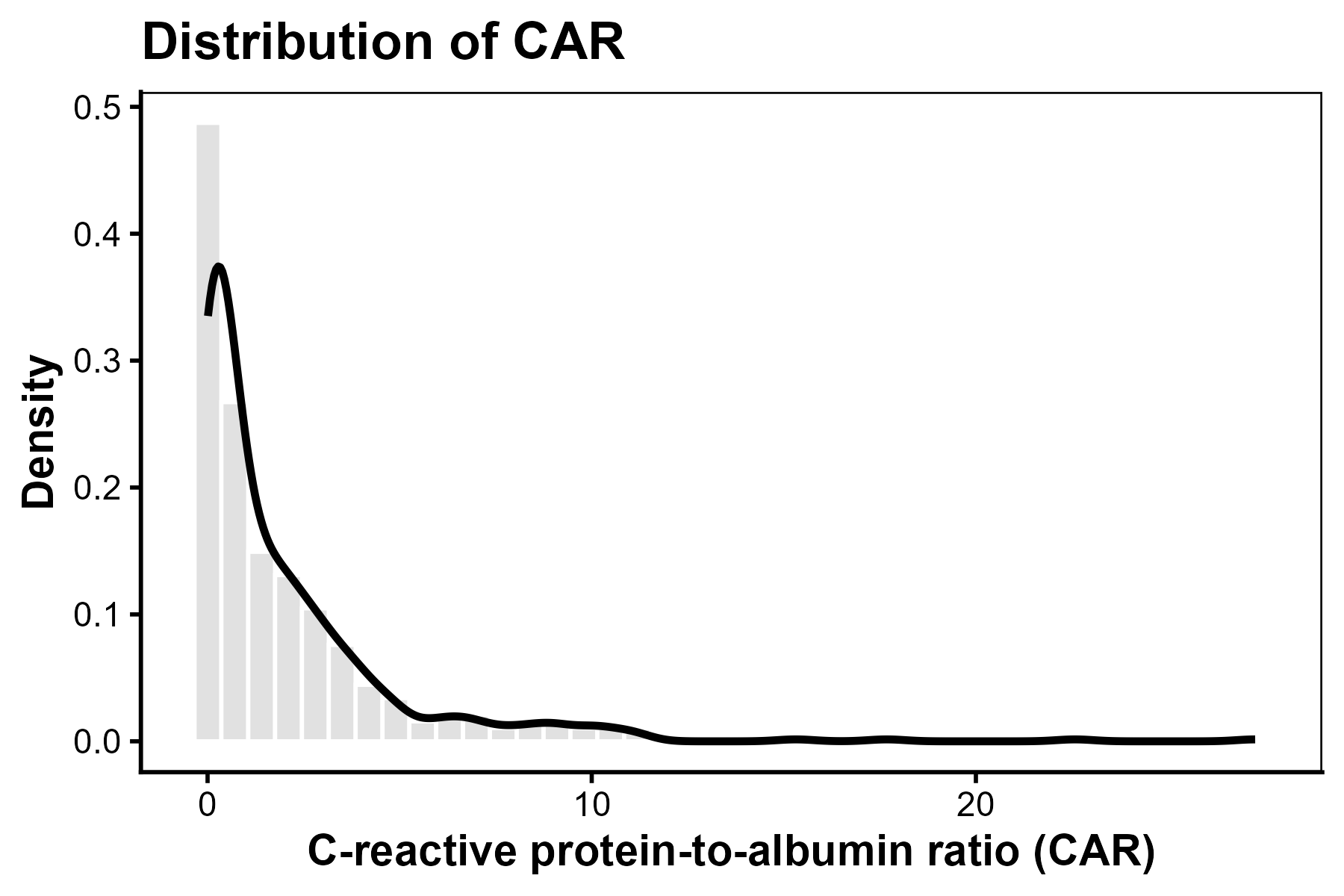
**

Histogram with an overlaid density curve illustrating the right-skewed distribution of baseline CAR in the study cohort. The long upper tail indicates a small proportion of patients with markedly elevated CAR values, supporting the use of sensitivity analyses to assess the potential influence of extreme values.
**Abbreviations:** CAR indicates C-reactive protein–to–albumin ratio.

**Supplementary Figure S3.** Boxplot of the C-reactive protein-to-albumin ratio.

**
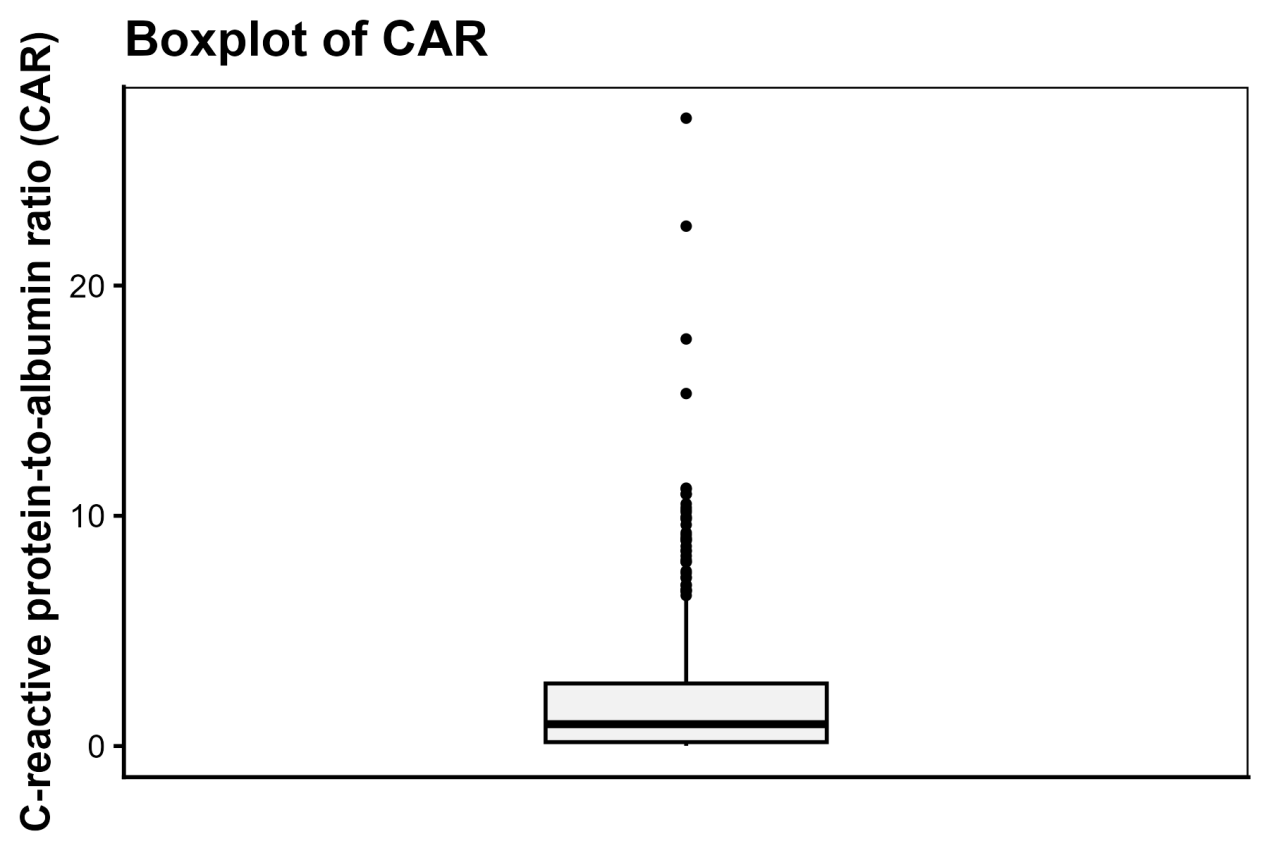
**

Boxplot of baseline CAR demonstrating the presence of high-end outliers, consistent with the right-skewed, long-tailed distribution observed in Supplementary Figure S1. This visualization supports the use of winsorization-based sensitivity analyses to evaluate the potential influence of extreme values on model estimates.
**Abbreviations:** CAR indicates C-reactive protein–to–albumin ratio.

**Supplementary Figure S5.** Restricted Cubic Spline Analyses of the Association Between the C-Reactive Protein–to–Albumin Ratio and All-Cause Mortality Conducted as Sensitivity Analyses


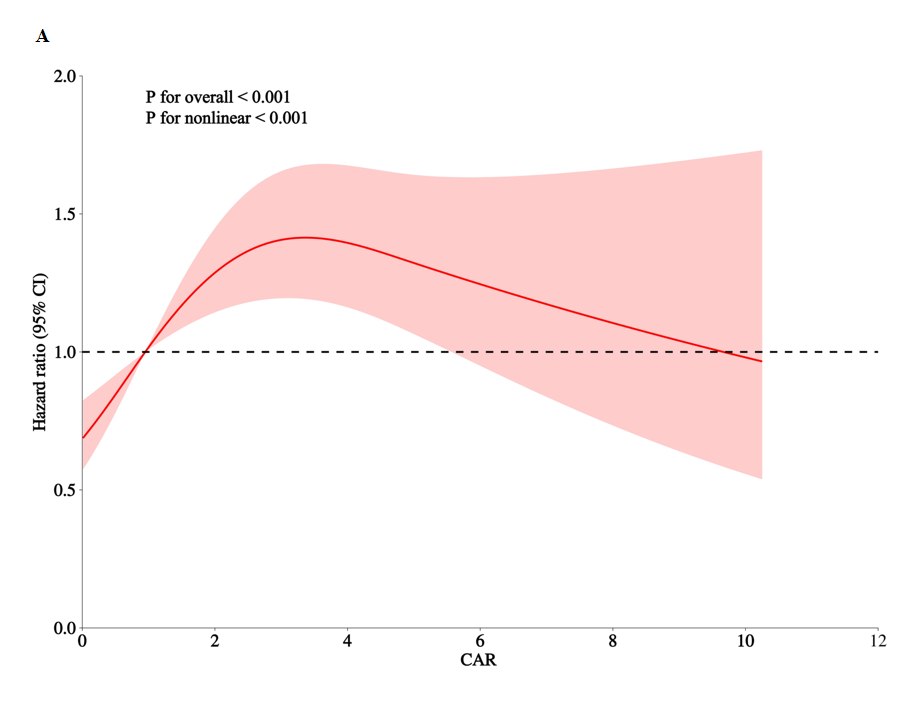


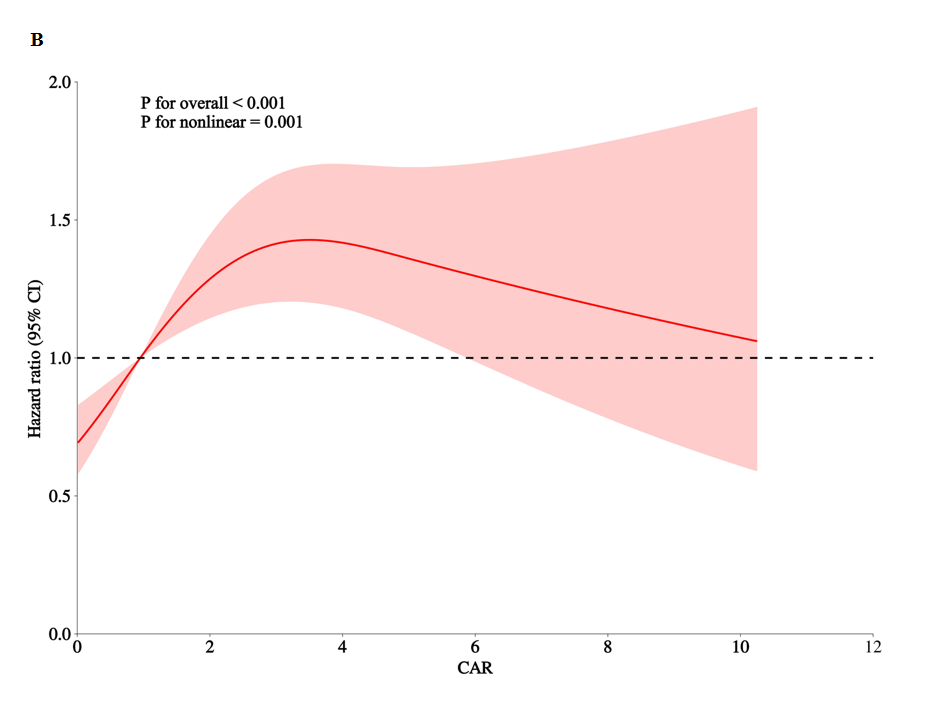


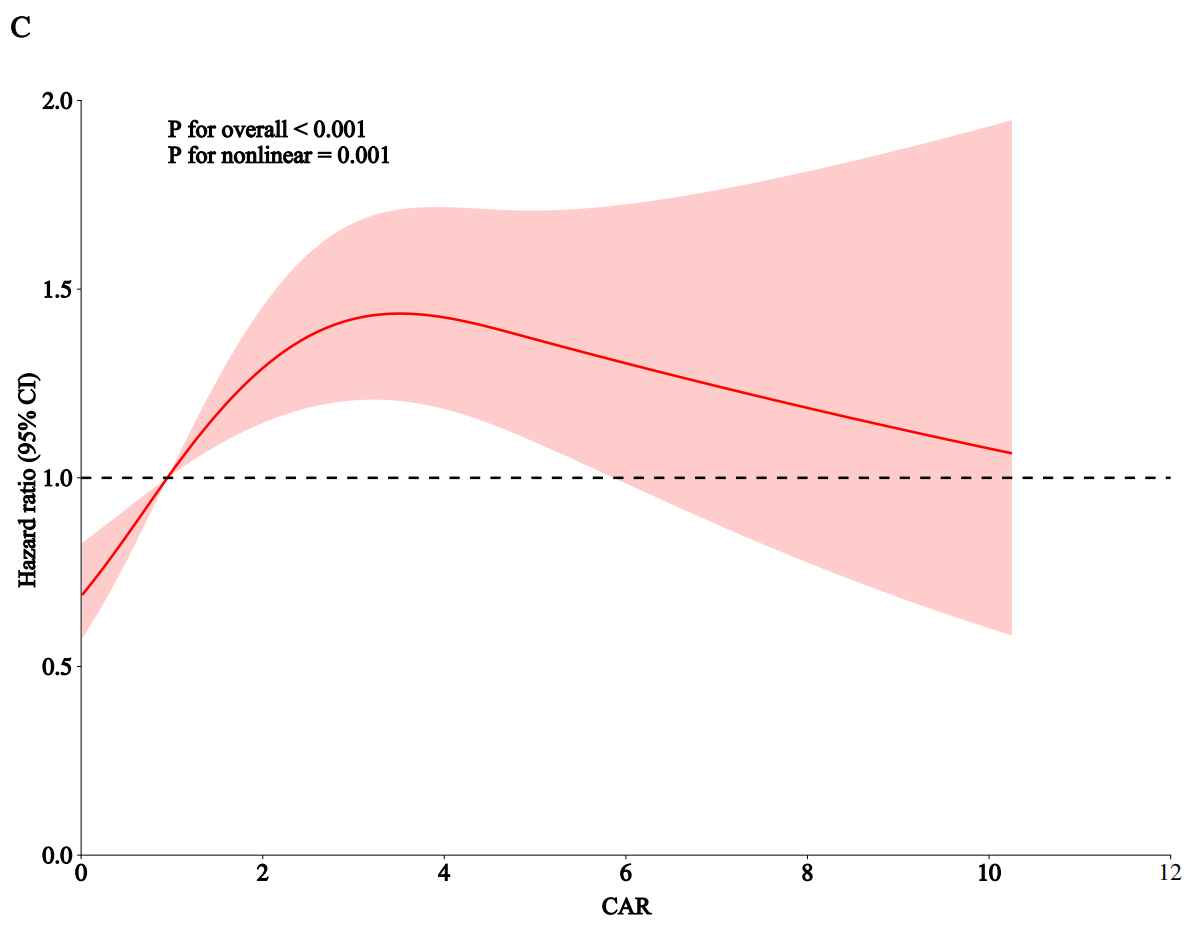


Restricted cubic spline (RCS) curves depicting the association between the C-reactive protein–to–albumin ratio (CAR) and all-cause mortality.
(A) Unadjusted model. (B) Model adjusted for age and sex. (C) Model adjusted for age, sex and comorbidities. The solid line represents the estimated hazard ratio, and the shaded area indicates the 95% CI. The dashed line denotes HR = 1.0.
Knots were placed at the 5th, 35h, 65h and 95th percentiles of CAR. Results of the fully adjusted model are presented in Figure 3 (P overall＝0.001; P nonlinear<0.001).

**Abbreviations:** CAR indicates C-reactive protein–to–albumin ratio; HR, hazard ratio; CI, confidence interval.

**Supplementary Figure S6.** Sensitivity analyses of restricted cubic splines with different knot numbers for baseline CAR and all-cause mortality.


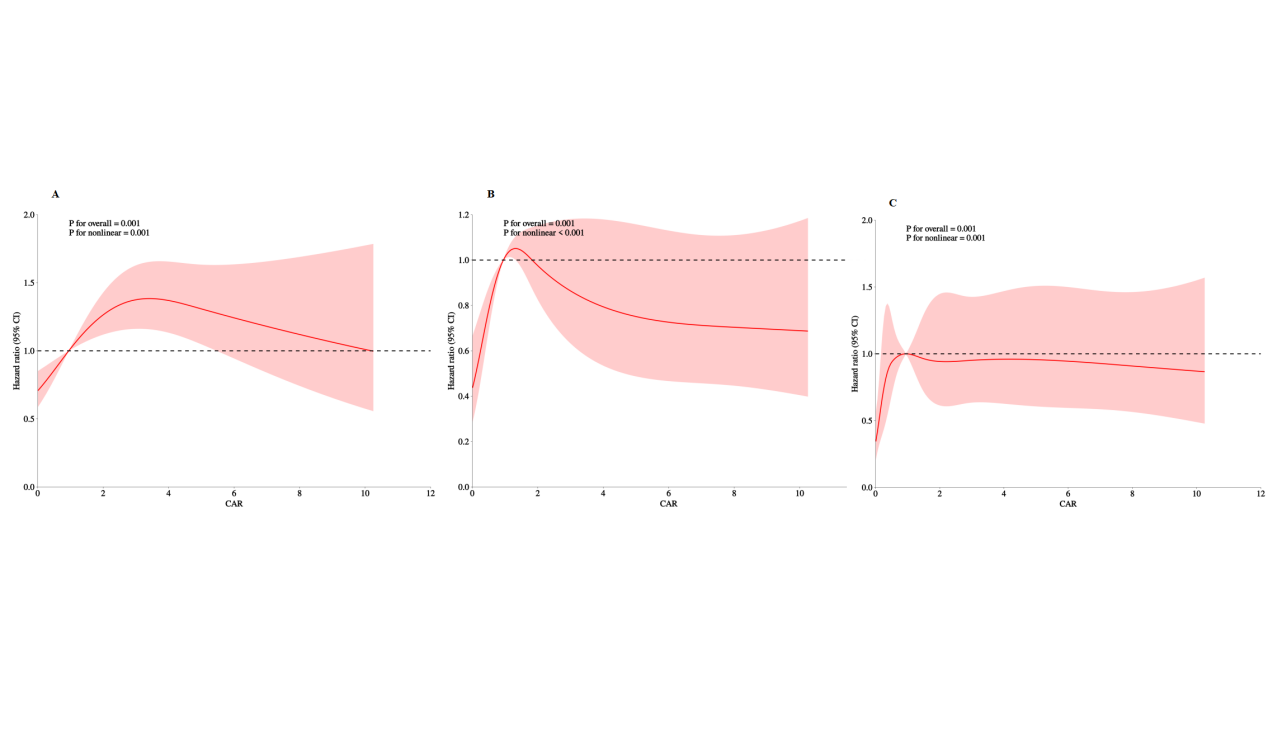


Restricted cubic spline curves for the association between baseline C-reactive protein-to-albumin ratio (CAR) and all-cause mortality were fitted within fully adjusted Cox models using (A) 3 knots, (B) 4 knots, and (C) 5 knots, with the median CAR as the reference. The overall nonlinear pattern was broadly consistent across knot specifications. The 3-knot model provided a more smoothed representation, whereas the 5-knot model showed greater local variability and wider uncertainty at the extremes, suggesting potential instability in sparse ranges. Therefore, the 4-knot model was retained for the main analyses. The dashed line indicates HR = 1.

**Abbreviation:** CAR, C-reactive protein-to-albumin ratio.

**Supplementary Table S6.** Threshold Effect of the C-Reactive Protein–to–Albumin Ratio on All-Cause Mortality in the Fully Adjusted Model

| **Model** | **Inflection point (CAR)** | **HR (95% CI) below threshold** | **P** | **HR (95% CI) above threshold** | **P** | **Likelihood-ratio P** |
| --- | --- | --- | --- | --- | --- | --- |
| Fully adjusted | 0.33 | 24.67 (16.66 - 125.21) | 0.010 | 1.00 (0.94 - 1.06) | 0.945 | 0.016 |

Segmented Cox regression assessing the threshold effect of the C-reactive protein–to–albumin ratio (CAR) on all-cause mortality in the fully adjusted model, which included age, sex, and comorbidities.
The breakpoint (CAR ≈ 0.33) was derived from the multivariable restricted cubic spline model.
Sensitivity analyses using less adjusted models yielded consistent patterns (data not shown).

**Abbreviations:** CAR indicates C-reactive protein–to–albumin ratio; HR, hazard ratio; CI, confidence interval.

**Supplementary Table S7.** Subgroup Analyses of the Association Between the C-Reactive Protein–to–Albumin Ratio and All-Cause Mortality

|  | **Case** | **Q1** | **Q2** | **Q3** | **Q4** | **P for trend** | **P for interaction** |
| --- | --- | --- | --- | --- | --- | --- | --- |
| Gender |  |  |  |  |  |  | 0.726 |
| Male | 308 | Ref. | 2.73 (1.52 ~ 4.91) | 2.55 (1.49 ~ 4.36) | 2.80 (1.63 ~ 4.84) | <0.001 |  |
| Female | 238 | Ref. | 1.90 (1.06 ~ 3.41) | 2.70 (1.49 ~ 4.88) | 1.96 (1.03 ~ 3.72) | 0.014 |  |
| Age (years) |  |  |  |  |  |  | 0.328 |
| <65 | 341 | Ref. | 3.35 (1.81 ~ 6.20) | 3.86 (2.09 ~ 7.13) | 3.50 (1.89 ~ 6.50) | <0.001 |  |
| ≥65 | 205 | Ref. | 1.66 (0.92 ~ 3.00) | 2.12 (1.22 ~ 3.67) | 1.90 (1.05 ~ 3.44) | 0.014 |  |
| Hypertension |  |  |  |  |  |  | 0.731 |
| No | 456 | Ref. | 2.26 (1.41 ~ 3.63) | 2.74 (1.72 ~ 4.37) | 2.49 (1.56 ~ 3.98) | 0.767 |  |
| Yes | 90 | Ref. | 1.43 (0.66 ~ 3.10) | 1.90 (0.90 ~ 4.00) | 1.67 (0.68 ~ 4.12) | 0.229 |  |
| Malignancy |  |  |  |  |  |  | 0.403 |
| No | 472 | Ref. | 1.94 (1.25 ~ 3.00) | 2.13 (1.37 ~ 3.30) | 1.94 (1.24 ~ 3.03) | 0.984 |  |
| Yes | 74 | Ref. | 2.64 (0.97 ~ 7.19) | 4.29 (1.70 ~ 10.79) | 3.88 (1.48 ~ 10.17) | 0.042 |  |

Hazard ratios (HRs) and 95% confidence intervals (CIs) for all-cause mortality according to prespecified subgroups defined by sex, age (<65 vs ≥65 years), hypertension, and malignancy status.
Analyses were conducted using multivariable Cox regression models adjusted for demographic and clinical covariates. Q1 served as the reference quartile.
P for trend values indicate linear trends across CAR quartiles within each subgroup, and P for interaction values indicate effect modification across subgroups.
No statistically significant interaction was observed (all P for interaction >0.05).

**Abbreviations:** CAR indicates C-reactive protein–to–albumin ratio; HR, hazard ratio; CI, confidence interval.
